# Effectiveness of a novel intervention (Super Rehab) in overweight patients with atrial fibrillation (SuRe AF): protocol for a randomised controlled trial

**DOI:** 10.1136/bmjopen-2025-103090

**Published:** 2025-09-14

**Authors:** David Murphy, John Graby, Theresa Smith, Oliver Peacock, Joanna Abramik, Charalambos Antoniades, Jonathan Carl Luis Rodrigues, Dylan Thompson, Ali Khavandi

**Affiliations:** 1 Cardiology, Royal United Hospitals Bath NHS Foundation Trust, Bath, UK; 2 Department for Health, University of Bath, Bath, UK; 3 Department of Mathematics, University of Bath, Bath, UK; 4 Department of Medicine, University of Oxford, Oxford, UK; 5 Radiology, Royal United Hospitals Bath NHS Foundation Trust, Bath, UK

**Keywords:** CARDIOLOGY, Exercise, Rehabilitation medicine

## Abstract

**Introduction:**

Atrial fibrillation (AF) is the most common sustained arrhythmia worldwide, associated with significant morbidity, mortality and healthcare utilisation. AF rhythm control strategies demonstrate attrition with time. A number of modifiable AF risk factors contribute to an atrial cardiomyopathy culminating in incident AF but importantly also recurrence. We propose that a novel multidisciplinary lifestyle intervention (Super Rehab, SR) may improve symptoms and AF burden.

**Methods and analysis:**

This is a single-centre, randomised controlled study. Patients aged ≥18 years with a body mass index ≥27 kg/m^2^ with paroxysmal or persistent AF will be randomised 1:1 to National Health Service (NHS) usual care (UC) or to SR (together with NHS UC). SR incorporates high-intensity exercise, personalised dietary advice and AF risk factor modification. SR will be undertaken over 12 months. In addition to baseline assessments, follow-up assessments will occur at the 6, 12 and 15-month time points. The primary outcome will be the difference in AF symptom burden at 12 months between groups. Secondary outcomes include AF burden (assessed by an implantable cardiac monitor), changes to cardiac structure and function and computed tomography-based assessment of epicardial adipose tissue.

**Ethics and dissemination:**

Ethics approval was granted by London-Chelsea Research Ethics Committee (reference: 22/LO/0479 22/08/2022). All participants will provide written informed consent prior to enrolment. Study findings will be disseminated via presentations to relevant stakeholders, national and international conferences and open-access peer-reviewed research publications. A summary will also be communicated to the participants.

**Trial registration number:**

ClinicalTrials.gov ID NCT05596175.

**Strengths and limitations of this study**

- Randomised controlled study examining the impact of a novel lifestyle intervention (Super Rehab) on atrial fibrillation (AF) in an National Health Service setting.
- Super Rehab combines 1:1 high-intensity interval training with resistance exercises and 1:1 dietary review sessions over a 12-month programme.
- Robust data on AF burden will be generated through the use of implantable cardiac monitors.
- Any cardiac structural and functional changes will be assessed with both echocardiography and CT coronary angiography.
- This is a single-centre study, which may affect its external validity.

## Introduction

 Atrial fibrillation (AF) is the most common sustained cardiac dysrhythmia occurring with increasing incidence and prevalence.^1 2^ It affects approximately 1.5 million people in the UK.^3^ AF imparts a significant health burden, most notably heart failure and stroke, in addition to conferring a 1.5–1.9-fold mortality r isk.^4^,^7^It is estimated that by 2040 AF direct care costs alone could constitute 4.3% of the entire National Health Service (NHS) budget.^8^

Current AF strategies targeting restoration of normal sinus rhythm include medications and interventional procedures such as direct current cardioversion (DCCV) and/or AF catheter ablation (CA).^9 10^ Current anti-arrhythmia medications demonstrate modest therapeutic benefit^11^ with safety considerations and side effect profiles often limiting their use.^11 12^ Despite the increasing incidence of AF, the introduction of novel anti-arrhythmic based therapy has not been forthcoming. DCCV requires a day case hospital admission and administration of anaesthetic. There is a significant recurrence rate with approximately 76% of patients returning to AF 1 year following DCCV.^13^ CA technology has improved with time^14^ but remains a costly invasive procedure.^15^ Overall complication and severe complication rates are approximately 4.5% and 2.5%, respectively.^16^ With a single AF ablation procedure, recurrence over longer-term outcomes (≥3 years) has been reported at 47%.^17^

There is increasing evidence that cardiometabolic risk factors are responsible for electromechanical alterations in the atrial myocardium leading to an atrial cardiomyopathy.^18^ This not only provides a mechanistic pathway to AF onset, but if not addressed, the substrate to recurrence, thereby representing a crucial target in the secondary prevention of AF. A system of solely targeting rhythm restoration without systematically treating the underlying drivers of AF is therefore sub-optimal.

### Arterial hypertension

Hypertension is present in 60%–80% of patients with established AF.^19^ Experimentally induced hypertension has been shown to cause left atrial hypertrophy, fibrosis and inflammation resulting in greater AF inducibility and duration.^20^ It confers a 150% greater chance of progressing from paroxysmal to persistent AF.^21^ Optimal blood pressure increases the freedom from AF following ablation.^22^

### Sleep disordered breathing (SDB)

Sleep disordered breathing (SDB) is highly prevalent in patients with AF.^23^ Hypoxaemia, hypercapnia and changes to sympathetic tone result in left atrial remodelling.^24 25^ AF-free survival following DCCV^26^ and AF ablation^27^ is reduced in those with SDB. Treatment of SDB reduces recurrence rates.^26^

### Hyperglycaemia

Electrical remodelling and myocardial fibrosis occur with hyperglycaemia.^28 29^ Poor glycaemic control prior to ablation is associated with a higher recurrence rate and indeed improvements in haemoglobin (HbA1c) pre-ablation have been shown to increase AF free survival.^30^

### Body mass index (BMI)

In the UK, one in four adults is obese, with a rising prevalence.^31^ Obesity increases left atrial size and epicardial adipose tissue volume (EAT).^32^ Such changes in the metabolically active EAT has been shown to result in the release of pro-inflammatory and pro-fibrotic mediators into the underlying myocardium.^32^ From a secondary care point of view, obesity has been shown to increase the recurrence rate of AF following catheter ablation,^33^ and weight loss prior to ablation improves freedom from AF.^34 35^

### Exercise and fitness

In addition to weight loss, improvements in physical fitness provide a synergistic effect. CARDIO-FIT (impact of cardiorespiratory fitness on arrhythmia recurrence in obese individuals with AF) demonstrated that an improvement of ≥2 metabolic equivalents conferred a 200% higher probability of AF-free survival over a 5-year follow-up period.^36^ Moreover, higher cardiorespiratory fitness is associated with a lower long-term all-cause mortality in patients with AF.^37^

### Previous work

Several studies have shown an improvement in AF burden and symptoms with weight reduction and risk factor modification.^38^,^40^ These studies however were predominantly cohort studies rather than randomised controlled trials and were undertaken in non-UK, non-NHS populations. There has been insufficient focus on combining supervised exercise and nutrition interventions together with risk factor modification. Our intervention will target a holistic approach to AF management with community-based personal trainers (PRs) and NHS dietitians. Additionally, much of the previous work has focused on patients at a tertiary referral centre at the time of an ablation rather than earlier in the referral process where we plan to recruit. The impact of an intervention on AF burden has also frequently been reported using intermittent ambulatory monitoring. This provides an incomplete assessment of overall AF burden. Our work will use an implantable cardiac monitor (ICM) for the duration of the study providing a detailed breakdown of the dysrhythmia burden.

### Aims and objectives

We aim to establish whether a structured cardiovascular rehabilitation intervention (Super Rehab (SR)), which targets AF risk factors, weight loss and greater physical fitness, leads to an improvement in symptoms for patients with AF in whom a rhythm control management strategy has been chosen.

### Primary objectives

1. To assess the difference in AF symptom burden (score range 0–35), as defined by the University of Toronto AF severity scale (AFSS), between usual NHS usual care (UC) and SR groups at 12 months. Additional evaluations will be conducted and reported at the 6- and 15-month time points.

### Secondary objectives

1. To assess the difference in % AF burden between groups at 12 months, with additional evaluations conducted and reported at the 6- and 15- month time points.
2. The assess the total duration and frequency of AF episodes between groups at 12 months, with additional evaluations conducted and reported at the 6- and 15-month time points.
3. To assess time to first AF recurrence following a DCCV or ablation (where ICM data is available prior to intervention)
4. To assess the change in the following cardiac and metabolic parameters between groups at 12 months, with additional evaluations conducted and reported at the 6- and 15-month time points:

- Weight, body mass index (BMI), waist circumference
- Cardiorespiratory fitness
- Visceral fat
- Left atrial size and strain.
- Peri-atrial fat volume and atriomic signal.
- Systolic and diastolic blood pressure
- Low density lipoprotein, high density lipoprotein, total cholesterol, glycated HbA1c and C-reactive protein
- % of time active.

5. To assess the difference in the following patient-reported outcomes between groups at 12 months, with additional evaluations conducted and reported at the 6- and 15-month time points:

- European Heart Rhythm Association (EHRA) scale
- Atrial fibrillation effect on quality of life (AFEQT)
- EuroQol-5 dimensions-5 levels (EQ-5D-5L)
- Hospital Anxiety and Depression Scale (HADS)
- Holistic capability assessment using the ICEpop CAPability measure for Adults (ICECAP-A).
- NHS resource-use questionnaire.

## Methods and analysis

### Study design

Single centre two-armed randomised controlled trial. Patients will be randomised (1:1) to UC or SR plus UC.

### Sample size

We aim to recruit 36 patients. The sample size was estimated using a one-tailed two independent sample t-test with a standardised effect size of 0.93 (estimated using a previous publication in a similar population using the same assessment methodology)^40^ with an alpha of .05 and power of .80 producing a sample size of 30 to detect a difference in AF symptom score. A 20% drop-out rate has been factored in to give a total sample size of 36 randomised 1:1.

### Study population and setting

Consecutive patients aged ≥18 years with either paroxysmal AF or persistent AF (defined as AF <1 year in duration at the time of referral to secondary care) will be prospectively identified at the Royal United Hospitals (RUH) Bath NHS Foundation Trust, a 750-bed district general hospital. Regional data demonstrated that over half (56%) of the local population are overweight/obese, with incidence rising in all communities.^41^ The RUH is aligned with current NICE guidance regarding the management of AF.^9^

### Patient identification and screening

Initial prescreening will prospectively identify patients referred for an ablation and/or DCCV through routine clinical care. Potential participants will be informed of the study protocol in writing and invited for an appointment at the study site. A clinician will then complete a face-to-face appointment where screening will be completed based on the inclusion and exclusion criteria outlined below.

### Inclusion criteria

1. Symptomatic paroxysmal or persistent AF.
2. Aged ≥18 years.
3. Patient has been referred for AF CA and/or DCCV as a result of routine clinical care.
4. BMI ≥27 kg/m^2^

### Exclusion criteria

Participants will not be eligible if they have conditions that preclude high-intensity exercise, including:

1. Significant coronary artery disease, defined as left main stem >50% stenosis and/or ≥moderate stenosis in ≥3 major epicardial vessels requiring revascularisation.
2. Unstable angina.
3. Heart failure with severe left ventricular systolic dysfunction (ejection fraction ≤35%) and/or New York Heart Association Class III/IV heart failure symptoms.
4. Significant cardiomyopathy (as assessed by a cardiologist).
5. Severe valvular heart disease.
6. Severe hypertension (BP >180/120 mmHg) despite anti-hypertensive treatment.
7. Uncontrolled arrhythmia or higher degree of heart block.
8. History of aortic dissection.
9. Recent acute pulmonary embolus, deep vein thrombosis, stroke or transient ischaemic event (<6 months).
10. Severe autonomic or peripheral neuropathy.
11. Significant acute or chronic renal failure that would preclude contrast use at CT.
12. Significant pulmonary fibrosis or interstitial lung disease (as assessed by a respiratory physician).
13. Physical inability to participate in high-intensity exercise.
14. Pregnancy or breastfeeding (due to the additional use of CT).
15. Prior AF ablation.
16. A clinically significant ECG abnormality, including but not limited to: sustained ventricular tachycardia, high-grade atrioventricular block (second-degree Mobitz type II or third-degree heart block), evidence of acute ischaemia (ST-segment elevation or depression >1 mm) or other arrhythmias deemed clinically significant by the study cardiologist.
17. Participation in another rehabilitation research study.
18. Inability to fully understand the instructions provided to them during the study.

Potential participants meeting the above criteria who are interested in joining the study will then be asked to provide informed consent for involvement in the trial. A copy of the consent form has been included in the supplementary material.

### Baseline assessments

**Figure 1:**
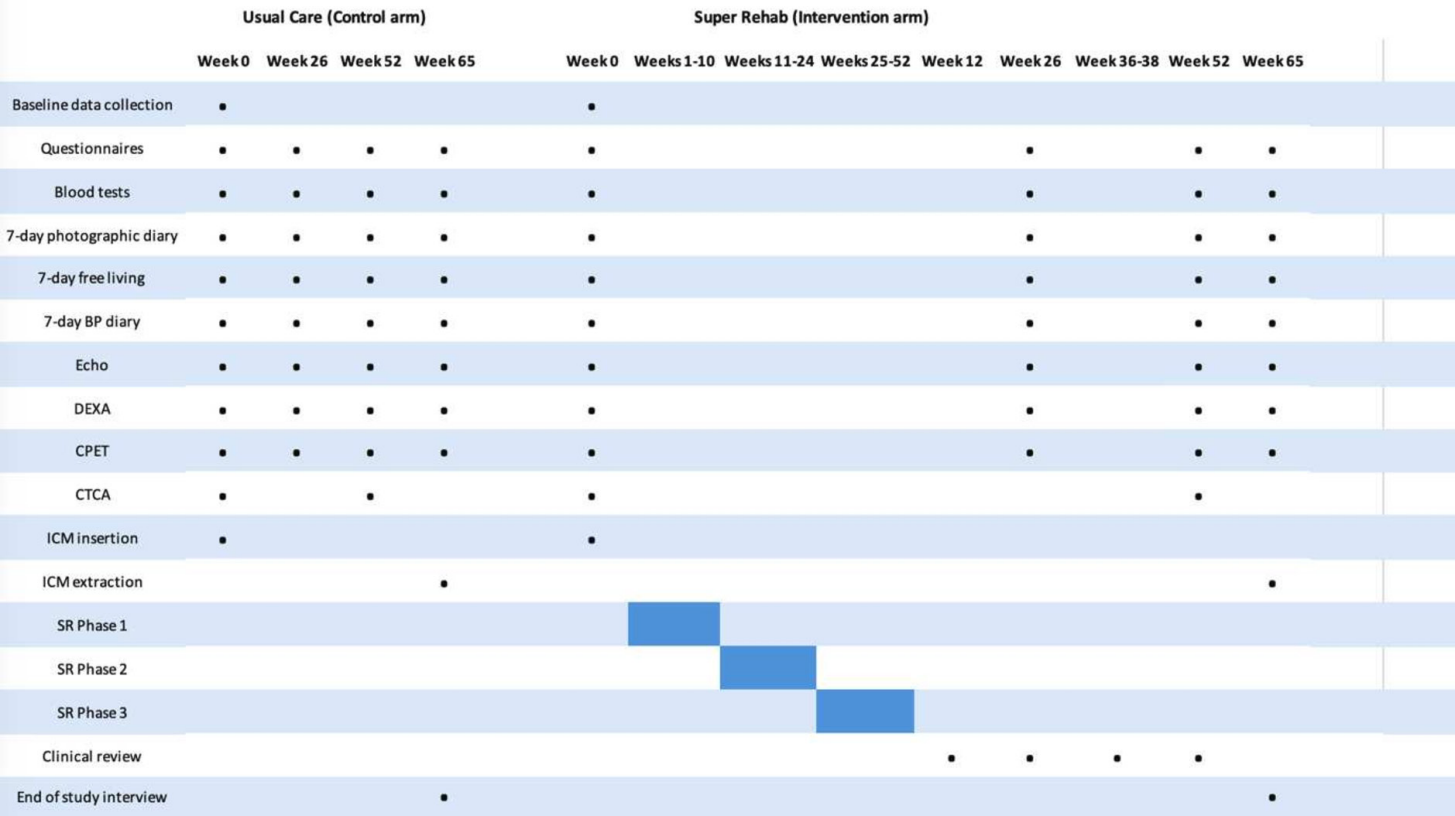
Study assessment and intervention timeline. Baseline, 6-month, 12-month and 15-month assessments include questionnaires, blood tests, echocardiogram, CPET, DEXA, 7-day photographic food diary, BP diary and GeneActiv monitoring. CTCA is carried out at baseline and 12 months. ICM is inserted at baseline and extracted at 15 months. Abbreviations: BP, blood pressure; CPET, cardiopulmonary exercise test; CTCA, CT coronary angiography; DEXA, dual-energy X-ray absorptiometry; ICM, implantable cardiac monitor; SR, Super Rehab.

Following consent to participate in the study, the following baseline data will be obtained (figure 1):

1. Medical history including medication list
2. Anthropometrics including: height, weight, BMI, abdominal waist and hip circumference
3. HbA1c
4. Seven-day photographic diet diary
5. Seven-day blood pressure diary (each patient will be supplied with a home blood pressure monitor).
6. Seven day free living activity and energy expenditure: GeneActiv monitoring device
7. Body composition: dual-energy X-ray absorptiometry (DEXA).
8. AF symptom burden: AFSS questionnaire and EHRA scale
9. Impact on quality of life: EQ-5D-5L and AFEQT
10. Psychological well-being assessment: HADS, ICECAP-A
11. NHS resource-use questionnaire

All consented participants will also have a baseline (1) transthoracic echocardiogram (TTE) assessing cardiac structure and function, (2) a CT coronary angiogram (CTCA) assessing both the coronary arteries and peri-atrial fat.^42^ Any severe valvular abnormalities and/or significant coronary artery disease diagnosed through these tests may preclude the participant from further involvement in the study, as outlined above.

In addition, a supervised symptom-limited cardiopulmonary exercise test (CPET) will be carried out to measure baseline level of fitness and to establish the ability of consented participants to complete high-intensity exercise safely. The results will also help to guide the prescription of exercise intensity parameters for those randomised to the intervention. Participants unable to complete the CPET will discontinue involvement in the study on safety grounds.

The CPET will be conducted on a cycle ergometer, beginning with a 5-minute warm-up against zero resistance. A subsequent ramp progression is used with resistance increasing by 5–25 watts per minute (individualised to participant). The test discontinues at volitional exhaustion targeting a Borg rating of perceived exertion (RPE) of ≥17. A subsequent recovery phase lasts ≥3 min against zero resistance. Achievement of VO_2_ peak will be assumed if the following criteria are met:^43^

1. Respiratory exchange ratio ≥1.10,

*or*

2. Increment in VO _2_≤5 mL/kg^-1^.min^-1^ in response to increasing gradient.

*and*

3. RPE ≥17.

On successful completion of the baseline assessments, all participants will have an ICM inserted subcutaneously to monitor AF burden for the duration of the study (Biomonitor IIIm, Biotronik SE and Co. KG). Although ICM implantation is invasive, the procedure is low risk, and participants will be closely monitored throughout the study to minimise any potential complications. ICM has been used in numerous AF studies, and they represent the gold standard of rhythm monitoring. Additional informed consent will be obtained for the implantation procedure. The device will be removed on completion of the study.

### Randomisation

Participants will then be randomised to either UC or SR using a web-based platform (Sealed Envelope: https://www.sealedenvelope.com/) in permuted blocks of four using an allocation sequence generated at random. Stratification will be by AF subtype (paroxysmal or persistent). Participants’ general practitioners will be informed of their patients’ involvement in the study and will be provided with a study information sheet.

### Intervention and control groups

#### Usual National Health Service (NHS care/control arm

In addition to any management advice given to patients through their routine clinical care, all participants in the study will be given one-off lifestyle advice by a clinician within the research team recommending moderate intensity exercise, weight loss and reduction in alcohol intake in line with ESC guidance.^10^

#### Super Rehab (plus usual care)/intervention arm

**Figure 2:**
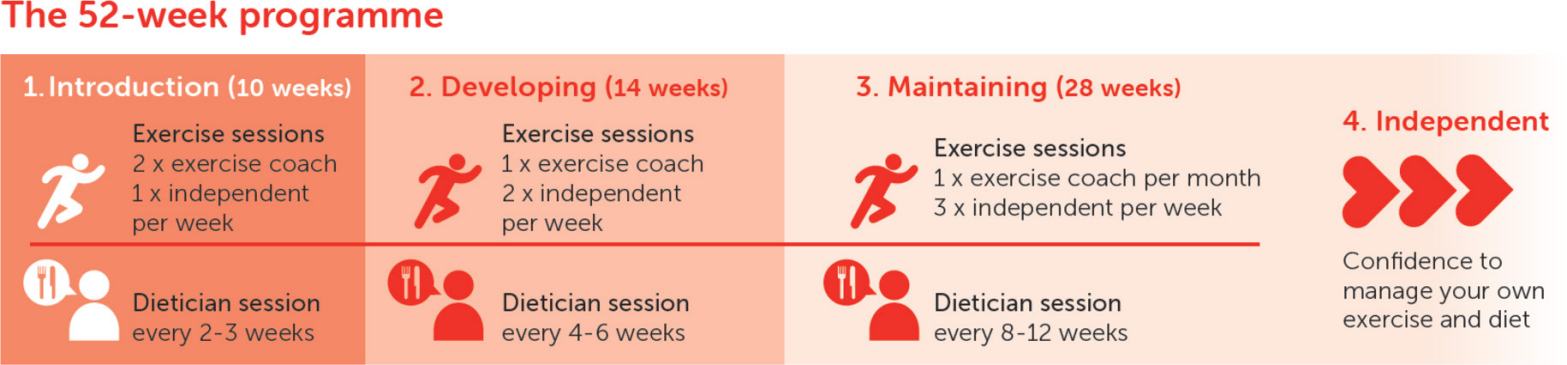
The Super Rehab programme.

A detailed description of the SR protocol is provided in the online supplemental file 1. In brief, SR consists of a 52-week rehabilitation intervention targeting positive dietary changes, weight loss and improved physical fitness in addition to identifying and treating any modifiable AF risk factors. The SR programme is overseen and coordinated by a cardiologist with concurrent multidisciplinary expertise offered by a dietitian, PT and an arrhythmia specialist nurse practitioner provided in a 1:1 manner. Care is individualised with the aim of optimising the management of each participant’s AF. SR is divided up into three phases (1) *induction* (10 weeks), (2) *developing* (14 weeks) and (3) *maintaining* (28 weeks) with a graded reduction in the frequency of supervised sessions facilitating increased participant confidence and autonomy (see figure 2).

1. *Dietetics*
  Once randomised to the intervention arm, participants will receive a SR introductory educational booklet which includes the aims of SR in addition to structured dietary and exercise advice. A dietitian will work with participants using photographic diet diaries to identify areas for improvement. They will provide tailored advice through the study to identify and remove any residual barriers to dietary improvements. Dietitian sessions will take place either face-to-face or remotely depending on participant preference.
2. Exercise
  Participants will undertake both supervised (with a PT) and homework-based exercise sessions. PT supervised sessions will consist of resistance training and high-intensity interval training using the Norwegian 4×4 model.^44^ Baseline CPET results for each participant will help provide guidance to set exercise intensity parameters. The PT will additionally prepare suggested homework exercises. PT-led sessions will be undertaken at a local gym facility.
3. AF and risk factors
  A 3-monthly clinical review delivered by an arrhythmia specialist nurse will aim to improve cardiospecific AF risk factors including lipid targets, glycaemic control and blood pressure control. SDB will be screened for and referred for further review as necessary. AF rhythm control strategy will be reviewed and heart rate controlled in line with guidance. Clinical review sessions will take place remotely.

### Assessments

In addition to baseline measurements detailed above, the following assessments will be carried out at 6, 12 and 15 months (see figure 1).

1. Medication list
2. Anthropometrics
3. Fasting blood tests.
4. Seven-day photographic diet diary.
5. Seven-day blood pressure diary
6. Seven-day free living activity.
7. AFSS questionnaire and EHRA class
8. EQ-5D-5L, AFEQT, HADS, ICECAP-A and NHS resource use questionnaires.
9. TTE
10. DEXA
11. CPET
12. A repeat CTCA with peri-atrial fat analysis will be carried out at 12 months only.

### Study status

Recruitment for the study began on 3/4/23. At the time of manuscript submission, recruitment has been completed with follow-up and participant involvement still ongoing. The study is expected to complete final data collection by November 2025.

### Data management and data analysis plan

#### Data management

All data will be stored and analysed pseudonymously using a unique identifier code. An electronic cross-referencing list will be stored on an NHS password-protected computer database at the study site. Only pseudonymised data using the unique identifier code will be used on datasets shared with external collaborators. All research records will be destroyed after 10 years.

Peri-atrial assessment by CTCA used in this study requires images to be transferred off-site to the Oxford Translational Cardiovascular Research Group core lab at the University of Oxford. We will use the existing pathway for pseudonymised image transfer via the study sponsor’s collaboration with the University of Oxford on The Oxford Risk Factors And Non-Invasive Imaging Study,^45^ which has separately been approved by the South Central - Oxford C - Research Ethics Committee (Reference 15/SC/0545). Existing secure information sharing protocols for standard clinically indicated scans will be used for image transfer.

### Data analysis

The distributional properties of the continuous variables will be examined by plots. Participant baseline characteristics and health outcomes will be summarised (including missing data) using descriptive measures: mean (SD) or median (IQR) for symmetric or skewed continuous variables, respectively; number (percent) for categorical variables; and mean (SD) absolute and percent change for longitudinal data will be reported separately by treatment allocation.

The primary outcome, change in AF symptom score, will be assessed using a two-way analysis of covariance (ANCOVA) adjusted for baseline score and AF type using data from subjects where both baseline and follow-up data are complete. The impact of missing follow-up data on the primary analysis will be explored with sensitivity analysis using methods such as last observation carried forward. This approach is likely to provide a conservative estimate of treatment effects. A prespecified secondary analysis will include a two-way ANCOVA for change in AF symptom score at 6 months, again adjusted for baseline score and AF subtype. To aid interpretation, changes will be considered in light of the minimally clinically important difference (MCID). While an anchor-based MCID has not yet been formally established for the AFSS, changes of approximately 3–5 points are considered clinically meaningful in analogous AF-specific instruments, such as the AFEQT. This range will be used to contextualise the clinical relevance of observed changes.

For continuous secondary outcomes, two independent samples t-tests or non-parametric alternatives will be used. Parametric assumptions will be checked by reviewing univariate histograms prior to formal analysis. For categorical secondary outcomes, change in status at follow-up will be compared between groups using χ² or Fisher’s exact test. Time to AF recurrence and event-free survival curves will be with Kaplan-Meier assessment and differences between curves tested with the log-rank test.

### Study governance

The trial management group (TMG) will monitor progress. Any adverse events will be reported to the trial steering committee (TSC). The TSC will be consulted for advice as required and monitor study progress. They will meet at baseline and 12 months with any additional meetings organised on request by the TMG. The data monitoring committee will meet at study initiation and then meet to review unblinded data at 12- and 24-month time points, alongside any additional safety issues for the trial and relevant information from other sources and advise the TSC on whether there are any ethical or safety reasons why the trial should not continue. They will also determine whether any additional interim analysis of trial data should be undertaken.

### Patient and public involvement

The NHS Research and Development office for Bath, Swindon and Wiltshire has supported our patient and public involvement (PPI). We collected PPI feedback through BSW’s dedicated group ‘PARTCIPATE’ and consulted with another patient PPI group within the *Cardiologist’s Kitchen* and *CardioFITr* programmes (run by clinicians in Bath). Patient-facing material was amended based on their feedback and we also integrated sample dietetics/exercise sessions for those in UC at the end of the study if requested by the participant. The primary outcome of symptom burden was selected by the clinical team, as it is often the key driver in decision-making around rhythm-control strategy management, including escalation to invasive treatments.

## ethics and dissemination

Ethics approval was granted by London-Chelsea Research Ethics Committee (reference: 22/LO/0479, 22/08/2022) and participants will provide written informed 21 consent prior to any study-related activities. Study findings will be discussed with participants and other relevant stakeholders (commissioners, charities, clinicians and members of the public). Results will be presented at national and international conferences as well as submitted for publication in a peer-reviewed journal.

## Supplementary material

10.1136/bmjopen-2025-103090 online supplemental file 1
