# Supplementary material for: Effectiveness of a novel intervention (Super Rehab) in overweight patients with atrial fibrillation (SuRe AF): protocol for a randomised controlled trial
Source: BMJ Open. 2025 Sep 14;15(9):e103090. doi: 10.1136/bmjopen-2025-103090 (PMC12434741; doi:10.1136/bmjopen-2025-103090)
Supplement: online supplemental file 1 [file bmjopen-15-9-s001.docx]

**Supplementary material**

# **Overview**

The overarching purpose of the Super Rehab (SR) rehabilitation programme is to integrate multi-disciplinary care to provide a superior pathway for the treatment of cardiovascular disease. The programme focuses on exercise, weight loss, fitness, diet and modifiable cardiovascular risk factors. This is delivered concurrently with any pharmacological or interventional procedures required, with all elements overseen by a cardiologist. Sessions are delivered 1-to-1 and individualised with particular emphasis on enabling and supporting successful behavioural change for the participants in an effort to maximise health outcomes.

Super Rehab has three phases: (1) introduction, (2) developing and (3) maintaining (see Figure 1). The exercise component will be delivered wherever possible in local community-based exercise facilities, with the last phase offered virtually if required. The dietary component will either be face-to-face or virtually, depending on individual participant preference and the nurse led clinical interactions will be virtual.

Phase 1 – Introduction (10 weeks); Exercise: Twice-weekly 1-hour supervised exercise sessions (described below), with once-weekly sessions of prescribed moderate-intensity ‘homework’. Weekly body metrics (blood pressure, heart rate, body mass index [BMI]) will enable goal-setting and biofeedback to encourage adherence. Diet: 30-minute educational sessions every two weeks. These will be delivered virtually or built into exercise sessions, minimising patient travel.

Phase 2 – Developing (14 weeks); Exercise: Reduced supervised session frequency to once-weekly, with two sessions of prescribed moderate-intensity homework per week. Body metrics measured and fed back twice weekly. Diet: A 30-minute “touch-base” session once per month, providing ongoing behavioural support of self-monitoring and goals.

Phase 3 - Maintaining (28 Weeks); Exercise: Supervised sessions reduced to once monthly, with twice weekly prescribed sessions of homework (now higher-intensity aerobic exercise) and once weekly resistance exercise session. Body metrics assessed monthly. Participants will be offered the option of having the supervised sessions in this phase delivered virtually or continuing face-to-face. Diet: A 30-minute session twice monthly, responding to individual barriers and changes in behaviour, where necessary, to support ongoing engagement with nutritional strategy.

Figure 1. The Super Rehab programme.

# **Exercise component - supervised sessions:**

HIIT: Experienced trainers will lead supervised 1:1 exercise sessions incorporating high-intensity interval training (HIIT) based on the Norwegian 4x4 model.^[1]^ HIIT has been shown to reduce the time in AF for those without permanent AF, improve health related quality of life and represents a more time efficient option than more moderate intensity exercise.^[2,3]^ At the start of each session, participants will be asked about any change in symptoms or medications and have blood pressure and heart rate measured.

The initial session will consist of a graduated, lead-in of moderate-intensity exercise prior to commencement of HIIT in subsequent sessions. Participants will be taught the Borg Rating of Perceived Exertion ^[4]^(RPE; 6–20) to guide exercise intensity. HIIT will be performed on a static bike (or alternative cardiovascular equipment if unable to cycle) and will comprise a three-minute warm-up; four four-minute high-intensity intervals at RPE 15 (hard), finishing at RPE 17-18 (very hard); three minutes of active recovery at RPE 11-13 (somewhat hard) between each interval; and conclude with three to five minute recovery.

Trainers will help maintain target intensity and monitor participants throughout for concerning symptoms. Heart rate data, final RPE and duration for each high-intensity interval will be recorded for subsequent analysis. The exercise trainer will feedback data (e.g. total virtual distance cycled or power used) to participants as they work through the programme to highlight visible progress being achieved.

The goal as participants progress through the programme will be to gradually increase their overall workload (via watts or speed) on a weekly basis as they become fitter and stronger. To achieve this, trainers will gradually increase the bike’s resistance or participant’s cycle speed, using heart rate response to ensure they remain in their target zone as workload increases.

Resistance Exercises: This has additional benefits, especially for blood pressure. ^[5,6]^ Sessions will therefore conclude with twenty minutes of resistance training.

This will be based on a short series of circuit training and will rotate muscle groups between sessions to allow recovery and maximise benefit. During the introduction phase trainers will utilise gym resources (free weights and weight machines) to build strength and confidence. In the subsequent developing phase this will switch to resistance against own body weight and bands to ensure patients learn routines that can then be undertaken in their own home in the maintaining phase and beyond.

The specific exercises are not directly prescribed to allow trainers to identify and respond to the individual needs of each participant. They will though be asked to follow this guiding framework and to record the exercises performed:

Introducing: Many patients will be deconditioned; therefore initial sessions will begin with 10 – 15 repetitions at 40 – 50% of the 1 rep-max using free weights or weight machines. When ready (typically after four to five weeks), progress to 60 – 80% of the individual’s one repetition maximum.

Developing & Maintaining: Move to exercises against gravity/own body weight and use of exercise bands so patients should learn exercises they can start to use on their own. Circuits of 10 – 15 reps at 60 – 80% of the individual’s one repetition maximum.

# **Exercise component – homework Sessions:**

To enhance the weekly workload achieved and support long-term behaviour change, participants will be prescribed ‘homework’ exercise sessions. This will comprise moderate-intensity aerobic exercise (60 – 75% of heart-rate max) for 45-minutes in induction and consolidation phases (e.g., brisk walking), and increase to higher-intensity exercise (e.g., hill walking or jogging) in the maintenance phase. A once weekly resistance exercise session using workouts learnt in the supervised session during phases one and two will be added in phase three. Participants will be instructed as to how to monitor their symptoms, and a heart-rate monitor (MyZone) will be used to support self-directed exercise intensity at the appropriate level. This data will be reviewed at supervised exercise sessions to evaluate progress and provide support and kept for subsequent analysis.

# **Dietary component:**

The dietary intervention component comprises dietitian-led 1:1 consultation using evidence-based dietary advice. This approach has been shown to improve blood pressure, insulin resistance and glycaemic control, lipid profile, systemic vascular inflammation and BMI. ^[7-15]^

Patients will complete pre-session photographic diet diaries and will be provided with an educational booklet incorporating the key dietary messages and structure. Recognising that there is no ‘one-size-fits-all’ pattern, the dietitian will work with participants to identify residual barriers to dietary change and potential solutions. The photographic diet diary will help highlight incremental areas for improvement in a straightforward manner. The dietitian will take a patient centred approach to setting relevant short-term goals specific to the individual they are working with, which will be reviewed and built upon in each session.

The principles and guiding framework for the dietary advice will be:

- A focus on dietary patterns, including eating at regular mealtimes with portion control rather than “calorie counting”, snack reduction and identifying healthy alternatives.
- Reduced refined carbohydrate intake, avoiding starch-based vegetables and refined grains.
- Reducing added sugars, making use of diet diaries to highlight “hidden sugars” as well encouraging low glycaemic index foods in diabetic participants.
- Sensible drinking choices, recommending water over smoothies, diet drinks and juices, unsweetened tea/coffee and alcohol in moderation.
- Avoidance of ultra-processed foods, such as highly processed meat and take-away meals.
- Choosing healthy sources of protein, encouraging plant-based foods such as pulses and nuts, regular oily fish, low-fat dairy products, eggs and lean unprocessed cuts of meat (if desired).
- Avoidance of ‘low-fat’ products, in favour of natural, whole foods with unsaturated and/or mono-saturated fats.
- Educate patients on the importance of swapping saturated fats for mono- and polyunsaturated fats. Examples include identifying approaches to increasing natural, whole foods containing unsaturated fats, encouraging nuts, seeds, oily fish, avocado and extra virgin olive oil.
- Increased dietary fibre, focusing on achieving this via diversifying vegetable intake, which encourages satiety.
- Salt restriction.

Where indicated, dietitians will also continue to highlight the importance of smoking cessation.

# **Behavioural support:**

All practitioners will be required to use the following behavioural support tools in the delivery of Super Rehab.

- Education: Practitioners will reinforce the positive health outcomes that can be achieved with the suggested exercise and dietary changes the programme aims to deliver (i.e., helping patients to understand what to do, and why they are being asked to do it). This will build on their Super Rehab booklet. Prescribed ‘homework’ exercises will be personalised to each individual participant, establishing how best they can incorporate this into their lifestyle.

- Problem-solving: Using the principles of a motivational interviewing, or person-centred approach, practitioners will support lifestyle changes by helping participants identify their own barriers or logistical issues to achieving their goals (e.g. money, family or work life, intolerances, time), and how they could overcome these. Example questions and techniques will be provided to trainers in their Super Rehab manual.

- Social Support: Sustaining long term change is more likely when people have support from their own networks. Participants will be asked to identify their key social support (e.g. partner, friend, child), and how they could help as part of problem solving. Dietitians will be encouraged to recommend participants bring their key social support with them to dietary review sessions, and it will also be suggested that participants perform their homework exercise with them. This will help encourage and support participants, increasing the potential to maintain this lifestyle change in the longer term.

- Goal setting & biofeedback: All participants will also be set a series of longer-term goals for their involvement in the programme, typically:

- 1. ≥10% weight loss with a target BMI of 18.5 – 24.9 kg/m^2^
  2. A normal abdominal waist circumference (<80cm women / <94cm men)
  3. Improvement in cardiovascular fitness.

However, during the in-programme review sessions practitioners will focus on the shorter term, achievable, relevant goals related to the actions they can take and focus on between sessions that will help them achieve these longer-term goals. E.g., a specific change in food type in their diet rather than the broader outcome of weight loss. There will be follow-up on whether these goals have been met in subsequent sessions. Super Rehab practitioners will follow the SMART goal-setting principles:

Specific – set a clear target/change for them to make (what, when & where)

Measurable – ensure it can be assessed at future visits

Achievable – ensure the goal is something you (and they) believe they can do

Relevant – select goals that ensure a step-wise improvement

Time-bound – set a realistic time-frame for them to achieve the goal(s) by

Changes in body metrics such as weight and abdominal waist circumference will be used within sessions to feedback on performance – both for positive re-enforcement and to supplement goal setting. The 1:1 nature of the sessions will be used to develop rapport, engender a working relationship and a level of accountability on the participant to deliver the changes asked of them.

# **Clinical support:**

Remote consultation with an advanced clinical nurse practitioner will take place every three months during the intervention. This review will follow a standardised approach which will target three themes: 1) AF risk factors identification and treatment, 2) AF symptom burden and treatment, 3) discussion around any barriers to success in the intervention.

1. AF risk factors

- Hypertension: A one-week blood pressure diary will be reviewed for each participant. Guideline recommended medications will be used to target a blood pressure of <130/80 mmHg.^[16]^
- Hyperglycaemia: Guideline recommended medications will be used to target an HbA1c <4% +/- onward referral to the endocrinology services as required.^[17]^
- Smoking: Smoking cessation will be encouraged/facilitated
- Dyslipidaemia: Guideline recommended medications will be used to target an LDL <1.8 mmol/L. Treatment decisions will be based on the presence or absence of CAD on the CT imaging carried out as part of the study.^[17]^
- Sleep disordered breathing (SDB) will be screened for and referral for sleep study requested as appropriate.
- BMI, waist circumference, physical fitness will be discussed

1. AF symptom burden and treatment

AF symptom burden will be assessed, and rate and rhythm control will be instituted as required with onward referral for further DCCV and/or ablation as deemed necessary.

1. Discussion around any barriers to success in the intervention.

The clinic appointment will act as an opportunity to discuss any difficulties with the intervention, barriers to success or key areas that require further focus.

**Computed Tomography Coronary Angiography (CTCA):**

A CTCA will be carried out at baseline and at 12 months.

Rate control will be employed with oral beta blocker/oral ivabradine (+/- intravenous beta blocker) with target heart rate <60 bpm. 800 mcg S/L nitrate will be used for diagnostic grade CT in accordance with RCR/BSCI guidance.

Protocol will be prospective, high-speed pitch single heart beat acquisition (FLASH mode) targeting diastole and if sub-optimal imaging quality, a prospective step-and-shoot acquisition using millisecond systolic timing depending on heart rate.

CTCA Studies will be acquired with a 128-slice CT scanner (Siemens SOMATOM Drive, Siemens Healthineers, Erlangen). Imaging protocol will involve a test bolus technique (12ml Niopam 340 at 6ml/sec) with subsequent full acquisition (60-80ml Niopam 340 at 6ml/sec). A slice thickness of 0.6mm, a pitch sequential scan feed of 34.5mm, rotation time 0.28s, and a tube voltage reference of 120kVp and 220 reference mAs with automated tube current modulation will be used.

Default CTCA reconstructions will be: cardiac field of view 0.6mm coronary vascular reconstruction kernel (Qr40, Siemens Healthineers), 0.6mm sharp vascular reconstruction kernel (Bv45, Siemens Healthineers) and 0.6mm raw axial reconstructions without automated smoothing between steps (Truestack I30f, Siemens Healthineers). All CTCAs will be reported clinically as per Coronary Artery Disease Reporting and Data Systems 2.0 (CAD-RADS) using syngo.via post-processing software (Siemens Healthineers, Erlangen). Reporting of scans will be done in a blinded fashion.

Imaging specifications set at the baseline scan will be matched at the follow-up 12 month scan.

**Transthoracic echocardiography (TTE)**

A TTE will be carried out at baseline, 6 months, 12 months and 15 month time points.

Images will be acquired on a GE HealthCare Vivid^TM^ E95. A standard minimum dataset TTE will be obtained at baseline as per the British Society of Echocardiography (BSE) guidelines ^[18]^ and any abnormalities followed up as per normal clinical practice. Follow-up testing at 6-, 12- and 15-month time points will measure interventricular septum thickness, left ventricular volume, ejection fraction, left atrial volume, mitral valve inflow (E and A wave, where present), mitral valve tissue doppler imaging and left atrial strain, where adequate imaging is available. Left atrial strain analysis will be done using automative software (Echo Pac software v 108.1.5; GE HealthCare). All analysis will be done blinded to the participants allocated group.

***“Super Rehab”: A novel approach to reverse atrial fibrillation? -* CONSENT FORM**

**Please initial box**

1. I confirm that I have read and understand the participant information sheet (Version 1.2, 26/07/2022) for the above study (IRAS ID 307544) and have had the opportunity to ask questions.
2. I understand that my participation is voluntary and that I am free to withdraw at any time without

giving any reason, without my legal rights being affected.

1. I agree to a screening which will involve a history, physical examination and 12-lead ECG.
2. I agree to members of the research team at the Royal United Hospitals Bath having access to my medical records to assess my eligibility for this study and record relevant aspects of my disease history for the subsequent data analysis of the study findings.
3. I understand that individuals from the Royal United Hospitals Bath or regulatory agencies may require access to my medical records to verify my participation and check procedures followed.
4. I agree to complete questionnaires on four occasions during my participation in the study – at the start, and after 6, 12 and 15 months.
5. I consent to having an implantable cardiac monitor inserted on the left side of my chest wall under local anaesthetic to enable the rhythm of my heart to be monitored throughout the study. I understand that this procedure involves small risks including bleeding, bruising and infection, and a separate consent form will be completed for this at the time of the procedure.
6. I give permission to have a blood test taken via venepuncture (with a needle) at the start, and after 6, 12 and 15 months of my participation in the study.
7. I give permission to have an approximately 30 minute ultrasound scan of my heart (echocardiogram) performed at the start, and after 6, 12 and 15 months of my participation in the study.
8. I give permission to have a CT scan of my heart at the start and after 12 months of my participation in the study. I understand that these CT scans (in addition to the DEXA scans below) use ionising radiation, which may cause cancer many years or decades afterwards. I understand we are all at risk of developing cancer during our lifetime, with 50% of the population likely to develop one of the many forms of cancer at some stage during our lifetime and taking part in this study may increase the chances of this happening to me to about 50.10%.
9. I give permission for any unexpected abnormal findings on my CT scan to be discussed with relevant clinical specialties and my General Practitioner (GP).
10. I give permission to have a DEXA scan (which I understand involves a small dose of radiation) on four occasions during my participation in the study – at the start, and after 6, 12 and 15 months.
11. I give permission to have a cardiopulmonary exercise test (CPET) on four occasions during my participation in the study – at the start, and after 6, 12 and 15 months.
12. I agree to perform a home blood pressure diary (which I will record) on 4 occasions, at the start, and after 6, 12 and 15 months (and understand the blood pressure monitor will be provided for me).
13. To organise the date and time of the DEXA and cardiopulmonary exercise tests, I agree to researchers at the University of Bath having access to my name and contact details.
14. If selected, I agree to participate in Super Rehab and understand this will involve frequent high-intensity exercise (HIIT) sessions supervised by a trainer and dietary sessions with a diet diary with a nutritional advisor. I understand that studies have shown HIIT to be safe in patients with a history of atrial fibrillation, but that there is still a small risk of a cardiac event.
15. If selected to participate in Super Rehab, I agree for relevant personal information including my medical history can be shared with practitioners delivering the Super Rehab programme.
16. I understand that pseudononymised results (personally identifiable information replaced by my study ID) will be shared with other UK institutions (including the Universities of Bath and Oxford) and an industry partner (Biotronik).
17. I agree to take part in the above study.

Please initial either ‘Yes’ or ‘No’

**Optional additional clauses:**

1. I give permission to be contacted by the research team about future studies related

Yes

No

to my involvement in this study or related to my underlying medical condition.

1. I give permission for information obtained from this study to be used in future

Yes

No

studies if it can help answer important research questions. I understand that it might

not be possible to share the nature or findings of future research with me directly.

1. I give permission for my blood samples to be stored for the duration of this study or

Yes

No

until they degrade naturally, to allow further analysis of these samples for other future

studies if important research questions can be answered by using these samples.

No

1. I give permission for my tissue and personal data (blood, imaging, physical results,

Yes

etc.) to be retained by the research team in the unlikely event that I were to lose

capacity to consent during the research study.

1. I give permission for my tissue and personal data (blood, imaging, physical results,

Yes

No

etc.) to be retained by the research team if I elect to leave the study early.

1. I give permission for review of my clinical records for up to 5 years after the study.

Yes

No

1. If I decide to stop participating in the programme before the end, I agree to be

No

Yes

contacted by telephone to discuss my reasons and experiences of the programme.

________________________ ________________ ____________________

Name of Participant Date Signature

_________________________ ________________ ____________________

Name of Person taking consent Date Signature

1 for participant; 1 for researcher; 1 for medical records

# **References:**

1. Taylor J.L, Holland D.J, Spathis J.G, et al. Guidelines for the delivery and monitoring of high intensity interval training in clinical populations. Progress in Cardiovascular Diseases. 2019;62(2):140–146. doi: 10.1016/j.pcad.2019.01.004.
2. Reed J.L, Terada T, Vidal-Almela S, et al. Effect of High-Intensity Interval Training in Patients With Atrial Fibrillation: A Randomized Clinical Trial. JAMA Netw Open. 2022;5(10):e2239380. doi: 10.1001/jamanetworkopen.2022.39380.
3. Malmo V, Nes B.M, Amundsen B.H, et al. Aerobic Interval Training Reduces the Burden of Atrial Fibrillation in the Short Term: A Randomized Trial. Circulation. 2016;133(5):466-73. doi: 10.1161/CIRCULATIONAHA.115.018220.
4. Borg GA. Psychophysical bases of perceived exertion. Medicine and science in sports and exercise. 1982;14(5):377–81.
5. Braith R.W, Stewart K.J. Resistance exercise training: Its role in the prevention of cardiovascular disease. Circulation. 2006;113(22):2642–2650. doi: 10.1161/CIRCULATIONAHA.105.584060.
6. Schroeder EC, Franke WD, Sharp RL, Lee D chul. Comparative effectiveness of aerobic, resistance, and combined training on cardiovascular disease risk factors: A randomized controlled trial. PLoS ONE. 2019;14(1):1–14. doi: 10.1371/journal.pone.0210292
7. Unwin D.J, Tobin S.D, Murray S.W, et al. Substantial and sustained improvements in blood pressure, weight and lipid profiles from a carbohydrate restricted diet: An observational study of insulin resistant patients in primary care. International Journal of Environmental Research and Public Health. 2019;16(15). doi: 10.3390/ijerph16152680
8. Hoyas I, Leon-Sanz M. Nutritional Challenges in Metabolic Syndrome. Journal of Clinical Medicine. 2019;8(9):1301. doi: 10.3390/jcm8091301
9. Mach F, Baigent C, Catapano A.L, et al. 2019 ESC/EAS Guidelines for the management of dyslipidaemias: Lipid modification to reduce cardiovascular risk. European Heart Journal. 2020;41(1):111–188. doi: 10.1093/eurheartj/ehz455
10. Dong T, Guo M, Zhang P, Sun G, Chen B. The effects of low-carbohydrate diets on cardiovascular risk factors: A meta-analysis. PLoS ONE. 2020;15(1):1–16. 10.1371/journal.pone.0225348.
11. DiNicolantonio JJ, OKeefe JH. Added sugars drive coronary heart disease via insulin resistance and hyperinsulinaemia: a new paradigm. Open Heart. 2017;4(2):e000729. doi: 10.1136/openhrt-2017-000729.
12. Threapleton D.E, Greenwood D.C, Evans C.E.L, et al. Dietary fibre intake and risk of cardiovascular disease: Systematic review and meta-analysis. BMJ (Online). 2013;347(December):1–12. doi: 10.1136/bmj.f6879.
13. Forouhi N.G, Krauss R.M, Taubes G, et al. Dietary fat and cardiometabolic health: Evidence, controversies, and consensus for guidance. BMJ (Online). 2018;361:1-8. doi:10.1136/bmj.k2139.
14. Brandhorst S, Longo V.D. Dietary Restrictions and Nutrition in the Prevention and Treatment of Cardiovascular Disease. Circulation Research. 2019;124(6):952–965. doi:10.1161/CIRCRESAHA.118.313352.
15. Korakas E, Dimitriadis G, Raptis A, et al. Dietary composition and cardiovascular risk: A mediator or a Bystander? Nutrients. 2018;10(12). doi: 10.3390/nu10121912.
16. Task A, Members F, Hindricks G, et al. 2020 ESC Guidelines for the diagnosis and management of atrial fibrillation developed in collaboration with the European Association of Cardio-Thoracic Surgery ( EACTS ) The Task Force for the diagnosis and management of atrial fibrillation of the Europea. 2020:1-126. doi:10.1093/eurheartj/ehaa612.
17. Visseren F.L.J, Mach F, Smulders Y.M, et al. 2021 ESC Guidelines on cardiovascular disease prevention in clinical practice. Eur Heart J. Published online 2021:3227-3337. doi:10.1093/eurheartj/ehab484
18. Robinson S., Rana B., Oxborough D. et al. A practical guideline for performing a comprehensive transthoracic echocardiogram in adults: the British Society of Echocardiography minimum dataset. Echo Res Pract 7, G59–G93 (2020). doi:10.1530/ERP-20-0026
