# Supplementary material for: Effectiveness of a novel intervention (Super Rehab) in overweight patients with atrial fibrillation (SuRe AF): protocol for a randomised controlled trial
Source: BMJ Open. 2025 Sep 14;15(9):e103090. doi: 10.1136/bmjopen-2025-103090 (PMC12434741; doi:10.1136/bmjopen-2025-103090)
Supplement: Supplementary file 2 [file bmjopen-2025-103090.reviewer_comments.pdf]

# PEER REVIEW HISTORY

BMJ Open publishes all reviews undertaken for accepted manuscripts. Reviewers are asked to complete a checklist review form and are provided with free text boxes to elaborate on their assessment. These free text comments are reproduced below.

## ARTICLE DETAILS

### Title (Provisional)

Effectiveness of a novel intervention (Super Rehab) in overweight patients with atrial fibrillation (SuRe AF): protocol for a randomised controlled trial

### Authors

Murphy, David; Graby, John; Smith, Theresa; Peacock, Oliver; Abramik, Joanna; Antoniades, Charalambos; Rodrigues, Jonathan Carl Luis; Thompson, Dylan; Khavandi, Ali

---

## VERSION 1 - REVIEW

---

| | |
|---|---|
| **Reviewer** | **1** |
| **Name** | **Fukaya, Hidehira** |
| **Affiliation** | **Kitasato University School of Medicine, Cardiovascular Medicine** |
| **Date** | **20-Jul-2025** |
| **COI** | **None** |

---

General Comments

Murphy et al. present the protocol, detailing the design and rationale of the SuRe AF trial. This study is a single-centre RCT evaluating a multidisciplinary lifestyle intervention to improve atrial fibrillation (AF) burden and symptoms in overweight patients. The protocol is thorough and addresses a clinically relevant and timely topic. The integration of implantable cardiac monitoring, high-intensity interval training (HIIT), and nutritional support within a structured programme is a notable strength. However, several clarifications and refinements would enhance transparency and reproducibility.

Major Comments

1. The protocol plans to recruit 36 participants based on a one-tailed test with an effect size of 0.93. Given the exploratory nature and novelty of the intervention, a two-tailed test may be more appropriate unless a strong rationale for the one-tailed approach is provided. Furthermore, the justification of the effect size is based on a prior study (reference 40), but

details about its comparability in terms of population and design are not provided. Please clarify whether the referenced study used a similar outcome measure (AFSS).

2. As an open-label study, the potential for performance and ascertainment bias exists. While patient blinding is inherently difficult in behavioural interventions. Please specify whether outcome assessors (e.g., echocardiography, CTCA interpreters) blinded to group allocation? Please specify.

3. The control group is described as receiving “usual NHS care” and “one-off lifestyle advice.” Please clarify whether this advice is standardised, scripted, or documented in any way, as variability in delivery could affect outcomes.

4. All participants receive implantable cardiac monitors (ICMs), which is commendable for robust AF burden assessment. However, the protocol should include a brief justification of the ethical acceptability and safety monitoring of ICMs in patients who may not otherwise receive them.

5. The statistical analysis plan mentions descriptive handling of missing data but does not elaborate on imputation strategies or sensitivity analyses. A more detailed plan would strengthen the analysis section.

Minor Comments

1. The exclusion criteria are appropriate, but item 16 (“clinically significant ECG abnormality…”) is somewhat vague. Providing specific examples or diagnostic thresholds would be helpful.

2. The primary endpoint is the change in AF symptom score at 12 months. Including the minimally clinically important difference (MCID) for the AFSS would be helpful to contextualize findings.

3. Figure 1 provides a helpful visual timeline. It may help readers if key outcome assessments (e.g. CTCA only at 12 months) are explicitly indicated in the figure legend.

4. The PPI involvement is appropriately described and should be commended. Consider clarifying whether participants were involved in prioritising the primary outcome.

5. Page 14: There is a numbering inconsistency in the criteria for VO2 peak: points 6, 7, and 8.

6. Ensure consistent use of hyphenation throughout (e.g., “high-intensity interval training” vs. “high intensity interval training”).

---

## VERSION 1 - AUTHOR RESPONSE

Reviewer: 1
Major Comments
1. The protocol plans to recruit 36 participants based on a one-tailed test with an effect size of 0.93. Given the exploratory nature and novelty of the intervention, a two-tailed test may be more

appropriate unless a strong rationale for the one-tailed approach is provided. Furthermore, the justification of the effect size is based on a prior study (reference 40), but details about its comparability in terms of population and design are not provided. Please clarify whether the referenced study used a similar outcome measure (AFSS).

**We acknowledge the exploratory nature of the novel intervention, and our hypothesis and analysis are intended to guide future research in this area. A one-tailed test was pre-specified prior to study commencement for the sample size calculation based on a directional hypothesis that the multidisciplinary lifestyle intervention would reduce AF symptom burden. This decision was informed by prior studies (e.g., ARREST-AF, LEGACY) demonstrating improvements in AF symptoms with lifestyle modification. Given this consistent evidence base and the low likelihood of harm from this non-invasive intervention, we considered a one-tailed hypothesis appropriate in this context.**

2. As an open-label study, the potential for performance and ascertainment bias exists. While patient blinding is inherently difficult in behavioural interventions. Please specify whether outcome assessors (e.g., echocardiography, CTCA interpreters) blinded to group allocation? Please specify.

**Thank you we agree that the potential for performance and ascertainment bias exist, and we intend to mention these within the limitations section when reporting on the study. The 12-month intervention is structured in a phased manner such that frequency of contact is higher in the earlier stages. Symptom burden (together with secondary outcomes) will be assessed and reported for 6-, 12- and 15-month time points which allows us to evaluate whether reported effects are sustained beyond the period of most intensive participant contact. This may help to characterise the extent to which observed changes reflect true intervention effects versus attention-related influences. The reporting of serum biochemistry, echocardiography, CTCA and per-atrial inflammation will be assessed by reporters blinded to group allocation.**

3. The control group is described as receiving "usual NHS care" and "one-off lifestyle advice." Please clarify whether this advice is standardised, scripted, or documented in any way, as variability in delivery could affect outcomes.

**The one-off lifestyle advice is delivered by the same individual within the study; while not scripted, it aligns with current ESC guidance on lifestyle management for atrial fibrillation. This advice is provided in addition to usual care from the NHS clinical team overseeing the patient.**

4. All participants receive implantable cardiac monitors (ICMs), which is commendable for robust AF burden assessment. However, the protocol should include a brief justification of the ethical acceptability and safety monitoring of ICMs in patients who may not otherwise receive them.

**Included.**

**See pg14**

5. The statistical analysis plan mentions descriptive handling of missing data but does not elaborate on imputation strategies or sensitivity analyses. A more detailed plan would strengthen the analysis section.

**Included.**

**See pg19**

**“The primary outcome, change in AF symptom score, will be assessed using a two-way ANCOVA adjusted for baseline score and atrial fibrillation type using data from subjects where both baseline and follow-up data is complete. The impact of missing follow-up data on the primary analysis will be explored with sensitivity analysis using methods such as last observation carried forward (LOCF). This approach is likely to provide a conservative estimate of treatment effects. A pre-specified secondary analysis will include a two-way ANCOVA for change in AF Symptom Score at 6-months, again adjusted for baseline score and AF sub-type.”**

Minor Comments

1. The exclusion criteria are appropriate, but item 16 (“clinically significant ECG abnormality…”) is somewhat vague. Providing specific examples or diagnostic thresholds would be helpful.

**Amended.**

**See pg11**

2. The primary endpoint is the change in AF symptom score at 12 months. Including the minimally clinically important difference (MCID) for the AFSS would be helpful to contextualize findings.

**Included.**

**See pg19.**

3. Figure 1 provides a helpful visual timeline. It may help readers if key outcome assessments (e.g. CTCA only at 12 months) are explicitly indicated in the figure legend.

**Amended.**

**See pg14**

4. The PPI involvement is appropriately described and should be commended. Consider clarifying whether participants were involved in prioritising the primary outcome.

**Clarification included.**

**See pg20**

5. Page 14: There is a numbering inconsistency in the criteria for VO2 peak: points 6, 7, and 8.

**Adjusted.**

**See pg14**

6. Ensure consistent use of hyphenation throughout (e.g., “high-intensity interval training” vs. “high intensity interval training”).
**Changed to hyphenated throughout.**

*** ***
